# A Review on Flexible and Transparent Energy Storage System

**DOI:** 10.3390/ma11112280

**Published:** 2018-11-14

**Authors:** Jie Li, Qianqian Jiang, Nannan Yuan, Jianguo Tang

**Affiliations:** Institute of Hybrid Materials, The National Base of International Scientific and Technological Cooperation on Hybrid Materials, The National Base of Polymer Hybrid Materials in the Programme of Introducing Talents Discipline to Universities, College of Materials Science and Engineering, The Growing Base for State Key Laboratory, Qingdao University, Qingdao 266071, China; lijie19921113@163.com (J.L.); lynn051227@163.com (N.Y.)

**Keywords:** flexible, transparent, energy storage system, lithium-ion battery, super capacitor

## Abstract

Due to the broad application prospect, flexible and transparent electronic device has been widely used in portable wearable devices, energy storage smart window and other fields, which owns many advantages such as portable, foldable, small-quality, low-cost, good transparency, high performance and so on. All these electronic devices are inseparable from the support of energy storage device. Energy storage device, like lithium-ion battery and super capacitor, also require strict flexibility and transparency as the energy supply equipment of electronic devices. Here, we demonstrate the development and applications of flexible and transparent lithium-ion battery and super capacitor. In particular, carbon nanomaterials are widely used in flexible and transparent electronic device, due to their excellent optical and electrical properties and good mechanical properties. For example, carbon nanotubes with high electrical conductivity and low density have been widely reported by researchers. Otherwise, graphene as an emerging two-dimensional material with electrical conductivity and carrier mobility attracts comparatively more attention than that of other carbon nanomaterials. Substantial effort has been put on the research for graphene-based energy storage system by researchers from all over the world. But, there is still a long way to accomplish this goal of improving the performance for stretchable and transparent electronic device due to the existing technical conditions.

## 1. Introduction

With intensification of energy crisis, more and more countries and people are paying attention to the collection and application of renewable energy, such as wind energy, water energy and solar energy. Meanwhile, the exploration of stable new energy storage system has got increasing attention of scientific researchers. In order to find appropriate materials owing advantages with low cost, clean, flexible, transparent and high performance, or to design new structure to utilize this new energy. Lithium-ion battery [1,2,3,4,5,6] and super capacitor [7,8,9,10,11], as green energy, have advantages of large energy density, high power density, long service life, no environmental pollution and so on. So, a large amount of research on lithium-ion battery and super capacitor has been reported. In this paper, we will introduce the research and development of lithium-ion battery and super capacitor in recent years. Most of the previous energy storage devices are rigid which cannot meet today’s stringent requirements, because electrode materials are easy to be separated from the collector. Then influence electrochemical performance, even lead to short circuit and cause serious safety problems and great waste in bending and folding. Therefore, flexible and transparent energy storage system has been urgently used for portable wearable devices, light-emitting diode [12], transistor [13], energy storage smart window [7], gas sensor [14] and so on. Though the application of the flexible and transparent energy storage device like lithium-ion battery and super capacitor is an inevitable trend of the development, it still faces many challenges compared to the rigid devices. For example, a part of flexible energy storage device requires expensive raw materials. Even, some transparent devices have poor electrochemical performance, like short endurance. Moreover, it is hard to integrate flexibility and transparency together. Hence, it still has a long way to go for scientific research workers to fabricate new energy storage device integrated flexibility, transparency and with high performance.

In order to obtain excellent performance, extensive research especially for electrode has been studied. Due to excellent mechanical and electrical properties, nanostructure samples such as nanotube/nanowire, nanosheet and nanopaper have great potential to be fabricated as the flexible and transparent electrode [15,16]. For example, Ag nanowire networks with wavy configurations have been demonstrated. That networks show great potential in electromechanical stability based on transferring NW networks into a variety of substrates. Besides, the transmittance of Ag NW networks electrode reaches 80.1%, which means Ag NW networks are suitable for flexible and transparent device [17]. Nanopaper, a freestanding paper-like conductive film, is also used frequently because of unique features of superior integrated flexibility and transparency. Bin Yao and co-workers demonstrate molybdenum trioxide nanopaper as the ultrathin transparent paper electrode [18]. And this electrode exhibits excellent performance in both lithium-ion battery and super capacitor, indicating the great potential of this nanopaper electrode in flexible and transparent energy storage devices. Though the flexible or transparent device has been greatly developed, it still has a long way to develop non-polluted, high powered and low-cost new type energy storage system due to the limitation in the integration of flexibility and transparency.

## 2. Lithium-Ion Battery

### 2.1. Flexible Lithium-Ion Battery

**Figure 1:**
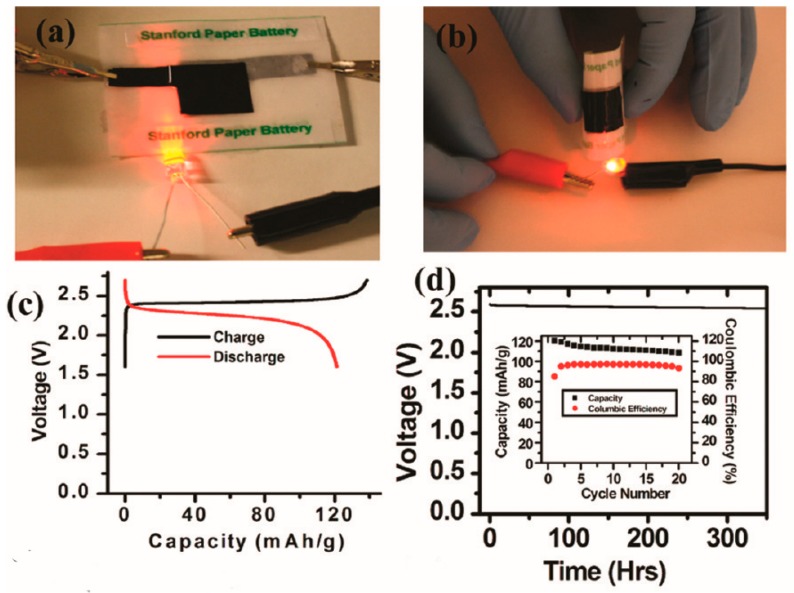
(**a**) Lithium-ion paper battery lighting red LED. (**b**) Flexible Lithium-ion paper batteries light an LED device. (**c**) Galvanostatic charging and discharging curves of paper battery. (**d**) Self-discharge behavior of a full cell after being charged to 2.6 V [23]. Copyright 2010, American Chemical Society.

Nowadays, our daily life is changed a lot, due to the development of electronic device but all of this equipment cannot be boot up without energy storage device. Lithium-ion battery as the most important one both in academia and industry occupies the vast majority of electronic device market [19,20,21]. It consists of cathode, anode, current collector, separator and packaging material. Lithium-ion battery, which was studied in the 80s of last century, has the advantages of high energy density, high output power, long cycle life, wide working range and relatively friendly to environment. Although the traditional lithium ion battery has many advantages, the disadvantages cannot be ignored. It is not only bulky but also unfolding. Even when the volume change is too large, it will lead to short circuit between cathode and anode, resulting in serious safety problems. Therefore, in order to adapt to the development of the next generation of flexible electronic equipment, the direction of the development of lithium ion batteries should also be developed in the direction of flexible and folding [22,23,24,25,26]. The usual method to fabricate flexible lithium-ion batteries is to coat active materials to flexible substrates. But, active material is easily separates from substrate by this method. In order to avoid this phenomenon, a one-step spraying method is adopt to prepare the embedded electrode materials within ultrathin carbon nanotube network for the flexible thin film lithium-ion battery, which can greatly improve the reversibility [1]. Owing to the shortened Li^+^ diffusion distance, sufficient conductivity, high contact surface area and excellent structure stability of the nanotube arrays, the self-supported Li_4_Ti_5_O_12_-C electrodes are expected to open up new opportunities to flexible electronic devices [27]. With the further research, various novel electrode nanostructures are proposed. A new-type integrated design of electrode is reported. Liangbing Hu and co-workers first use paper as separator and carbon nanotube thin films as both anode and cathode current collectors. Then, the lithium-ion battery is integrated into single sheet of paper by a simple lamination method. They find, the paper battery can continuously brighten the red LED for 10 min without fading (in Figure 1a). Even after bending the device manually to 6 mm 50 times, no paper battery failure is observed, suggesting excellent flexibility (Figure 1b). The first cycle voltage profile of lithium ion battery can be seen in Figure 1c. As shown in Figure 1d, after the first cycle, the coulombic efficiency is 94–97%. After 20 cycles, the discharge retention is 93%. The battery also displays good self-discharge performance in the inset of Figure 1d, which is indispensable for practical application [23]. In addition, graphene as a wonderful electrode material can be used in flexibly lithium-ion battery. It can composite with Bi_2_Se_3_to form Bi_2_Se_3_/Graphene (BSG) composite paper. Compared with the pure Bi_2_Se_3_nanosheets, this novel flexible BSG paper has a high reversible capacity of 203 mAh g^−1^ after 100 cycles at 50 Ma g^−1^, benefiting from graphene with outstanding electrical conductivity and excellent electrochemical discharge/charger stability [28]. The reasonable design of the next generation of activated energy storage graphene materials is demonstrated. The researchers found that three-dimensional graphene used for lithium-ion battery showed unexpected excellent performance. Na Li and co-workers fabricate a flexible graphene foam electrode lithium-ion battery with ultrafast charge and discharge rates [29]. The battery has ultrafast charge and discharge rates, due to graphene foam with excellent electrical conductivity and pore structure to enable rapid electron and ion transport, which indicates that 3D graphene, is available to be applied in a thin, lightweight and flexible lithium-ion battery with high-rate performance and energy density. 3D nitrogen-doped graphene foam with encapsulated germanium/nitrogen-doped graphene yolk-shell nanoarchitecture is fabricated, which has a high capacity values about 1220 mAh g^−1^, excellent cycling capability of over 96% reversible capacity retention after 1000 cycles and ultra-high rate capability due to the yolk-shell structure can effectively relieve volume expansion during the charge and discharge [30]. Moreover, it provides an effective thinking for those materials with a large expansion coefficient.

More significantly, the flexible lithium-ion battery full cell has been studied. Zongnan Deng and co-workers construct 3D ordered microporous MoS_2_@C nanostructure which is further fabricated as the flexible full cell. And the full cell shows outstanding area capacity and excellent capacity retention. The initial capacity of battery is 2.661 mAh cm^−2^ at 0.2 mA cm^−2^ current densities. The battery holds wonderful stability capacity retention after 20 cycles with the value of 1.681 mAh cm^−2^ at 5 mA cm^−2^ current densities, even with no capacity decay after another100 cycles. To test the stretchability, they demonstrated a stretchable LIB full cell. The brightness of the beginning is consistent with the brightness after the first bending and 300 bending cycles and there is no obvious change in brightness which illustrates the superior stability of the cell [31]. Through the above discuss, we can find that flexible lithium-ion battery has broad prospects in bendable smart phone, wearable electronics and medical devices, due to their special structures. So, it needs more attention to find suitable materials and design structure to improve flexibility, cycling stability and capacitance.

### 2.2. Transparent Lithium-Ion Battery

With the development of all transparent concept electronic devices, transparent electronic appears substantial applications for display, portable electronic device and solar cell [32]. For example, the concept of fully transparent cell phone has attracted great attention. Transparent screen also has rapid development. However, the key to develop transparent cell phone is to overcome the problem of full transparent battery. However, the research and development of transparent lithium-ion batteries is still at an initial stage. The typical method to make transparent device is to decrease the thickness of the battery, which is not suitable for the new type of battery.

It is well known that typical electrodes are always thick and most of them use the materials absorbing light, which will seriously affect the transparency of batteries. Hence, transparent lithium-ion battery faces a series of challenges. Francisco Martin and co-workers firstly describe the transparent electrode made from man-sized LiFeO_2_ directly grown on an indium tin oxide (ITO) substrate. The capacity of transparent electrode achieves 160 Ah kg^−1^ with capacity retention near to 98% [33]. And this is of great significance for the development of transparent batteries. But, such electrode has many limitations in the practical appliance of transparent batteries. For example, the transparency cannot meet the requirements of transparent devices. A novel grid-structured electrode breaks the limitations of changing the thickness of the film to improve transparency by aligning multiple electrodes together to increase amount of energy stored [34,35]. Yuan Yang and co-workers adopt a previously undescribed strategy of electrodes. A unique microfluidics-assisted method to pattern battery materials: (1) Transfer grid patterns from silicon mold to PDMS; (2) Evaporate gold current collector onto the PDMS substrate; (3) Fill in battery electrode materials by a microfluidics-assisted method and (4) Peel off gold film on top of the PDMS substrate. By this method, the transparency of battery is 78%, 60% and 30%, respectively, corresponding to the energy density of 5, 10 and 20 Wh^−1^ [35]. Certainly, high transparency device manufactured by this method require very high accuracy, which limits the more practical applications.

A fully integrated transparent battery obtained from a the rapid and controllable spin-spray layer-by-layer deposition is demonstrated [36]. These facile spin-spray layer-by-layer deposition films have a >87% transmittance, with ~2 nm/bilayer precision deposited on a transparent substrate. The capacity of full cell has an excellent stable over 100 cycles, with a capacity of 5 μAh cm^−2^. However, the limitation of the battery is inevitable that the full cell battery potential is lower than the initial cathode lithiation about 1 V. Though this battery exist limitation for the applications, the design direction of the new transparent battery is put forward. It provides a technology suitable for industrial production of transparent electrodes. Further, it can realize a potentially fully integrated transparent device. Therefore, the transparent lithium ion battery generated by this SSNL technology is an important topic in the future research of lithium ion batteries.

## 3. Super Capacitor

### 3.1. Flexible Super Capacitor

Super capacitor, called electrochemical capacitor or ultracapacitor, has gained increasing concerning. Compared with traditional capacitor, it has larger capacity, specific energy, capacity density, wider working temperature range and longer service life. Compared with batteries, it has higher specific power and environment friendly [37]. With the development of electronic device demands, super capacitor must be flexible and foldable, which will be a trend of the future development direction [38,39,40,41].

**Figure 2:**
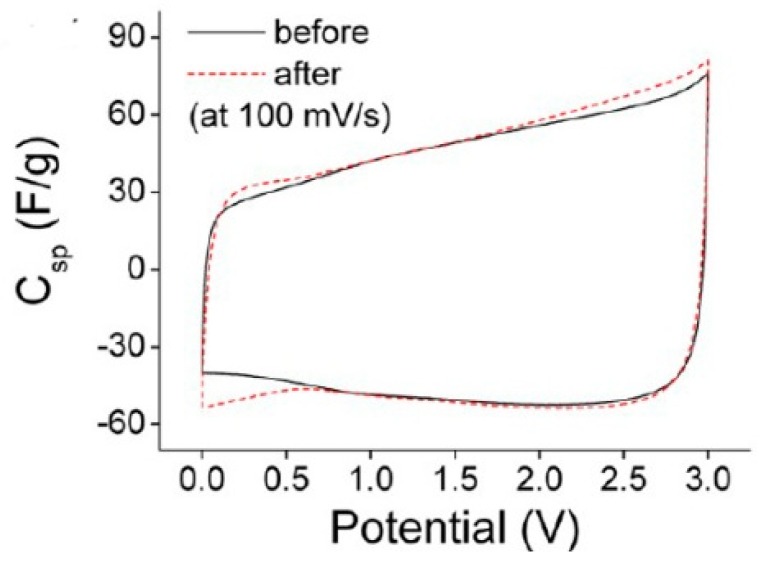
Cyclic voltammetry curves measured before and after 200 bending cycles [46]. Copyright 2012, American Chemical Society.

In general, super capacitor includes four components (electrode, separator, current collector and electrolyte) and each of components must be flexible. Only in that way, can the super capacitor meet the application requirements of flexibility. The typical and mature flexible super capacitor is a kind of super capacitor with two-dimensional planar structure. Carbon-based materials, mainly included carbon nanotube [42] and graphene [43,44,45], are the most widely used electrode materials in flexible super capacitor [10]. Compact carbon nanotube composites used as electrodes of super capacitor show high specific capacitance of 13.16 F cm^−3^ and excellent cycling stability even after 10,000 cycling. More importantly, the super capacitor displays outstanding stretch ability as high as 240% [42]. Papers are not only flexible but also can be readily integrated with carbon nanotubes. So, it is a great candidate for substrates of flexible super capacitor. For example, a flexible super capacitor fabricated with bacterial nanocellulose papers, carbon nanotubes and triblock-copolymer ion gels is reported. The super capacitors showed high tolerance against. As shown in Figure 2, it can highly retain through 200 bending cycles to a radius of 3 mm [46]. Besides, carbon nanotubes can also be combined with textiles. By coat single-walled carbon nanotubes (SWNTs) on cellulose and polyester fibers, we are taking a big step forward to wearable devices [47]. However, compared with other carbon-based materials, the specific surface area of carbon nanotube is too small, which hinder the energy density and power density of super capacitor. Due to the good electrical conductivity and large specific surface area, graphene has attracted much attention of researchers. 2D ultrathin MnO_2_/graphene nanostructure exhibits excellent electrochemical performance. The specific capacitance reaches 267 F g^−1^ at a current density of 0.2 A g^−1^ and the capacitance retention rate remains 92% after 7000 charge/discharge cycles. Even after repeated folding, the capacitance keeps >90% [43]. The rational design of planar super capacitor can make a great contribution to the future development of high performance, flexible energy storage device.

**Figure 3:**
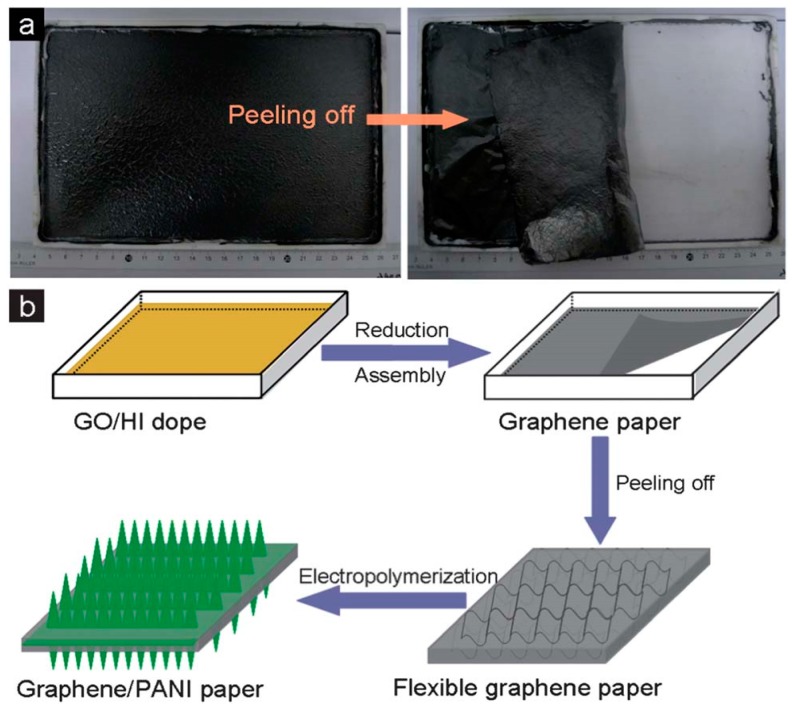
Schematic illustration of large-scale formation process graphene paper. (**a**) Peeling-off graphene paper with the size of 22 × 16 cmfrom a Teflon substrate. (**b**) Formation process of graphene–PANI paper [38]. Copyright 2013, the Royal Society of Chemistry.

On the other hand, polyaniline (PANI) is a very good super capacitor material, because it is easy to handle synthesis and has high specific capacitance and good environmental stability. So, the construction of a graphene-PANI composite electrode has been an attractive topic. Huai-Ping Cong and colleagues find a particular and facile manufacturing method on a large-scale free-standing graphene paper as shown in Figure 3. The photograph of graphene paper peeled off from Teflon substrate is shown in Figure 3a. Specific preparation process of graphene-PANI paper is displayed in Figure 3b. Firstly, the graphene paper formed by a one-step reduction-assembly method is peeled off from the Teflon substrate and then graphene paper is electro polymerized with the PANI nanorods to make the PANI nanorods uniformly distribute on the surface and interior of the graphene paper. Flexible graphene–PANI paper prepared by this method has the advantages of low cost, easy processability into devices and excellent energy storage performance [38]. Certainly, with the current technical backgrounds, carbon nanotube and graphene materials used in electrode are not very mature yet in industry. To meet the requirements of the micro and wearable electronic devices, a new type of super capacitor, one-dimensional fiber structure has been rapidly developed. Because of its small size in volume and light in weight, this kind of super capacitor not only has good flexibility but also has high portability [48], which makes it have broad application prospect in flexible super capacitor.

### 3.2. Transparent Super Capacitor

Super capacitor becomes more important recently owing to their high energy density, power density and great potential to act as integrated power sources for displays and smart windows. However, transparent super capacitor device with both good transparency and high specific capacitance is hardly reported, which poses a great challenge to develop high transparent super capacitor [49].

As an important part of super capacitors, any small progress in electrode can always arouse great interest of researchers. A transparent electrode material is made of ultrathin single wall carbon nanotube film. This ultrathin single-walled carbon nanotube film got high transparence approaching to 90% with decrease of the film thickness [50]. Besides, graphene has been often adopted on account of the unique planar structure, excellent electrical conductivity, large specific surface area and high optical transmittance. Transparent hybrid film electrode composed of reduced graphene oxide (RG-O) and Cu nanowires (NWs) not only has great transparency but also can improve electrical conductivity, oxidation resistance, substrate adhesion and stability of super capacitor in harsh environments [51].

**Figure 4:**
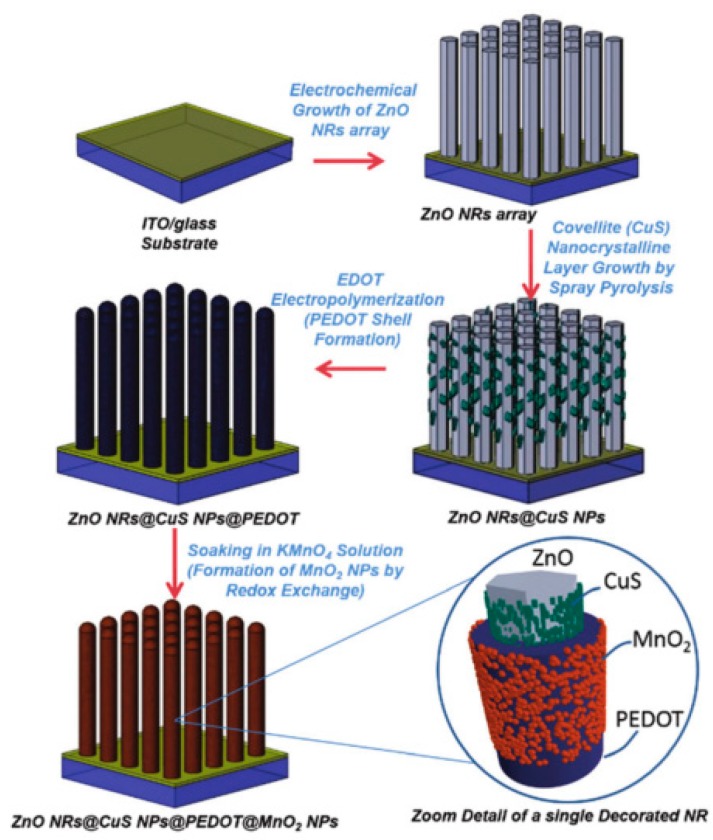
Schematic illustration of formation process of ZnONRs@CuS@PEDOT@MnO_2_ hybrid nanostructured electrode [49]. Copyright 2014, the Royal Society of Chemistry.

Besides, Jorge Rodrıguez-Moreno and co-workers introduce a semi-transparent super capacitor. The super capacitor has a novel architecture and hybrid electrode material with improved capacitive performance. And the schematic diagram of the vertically aligned ZnO@CuS@PEDOTcore@shellnanorods arrays decorated with MnO_2_ nanoparticles is demonstrated in Figure 4. The formation process of ZnO@CuS@PEDOTcore@shellnanorods arrays includes electrochemical growth of vertically aligned ZnO NRs array as nanostructure collector, CuS nanocrystal line layer grown by spray pyrolysis, the poly (3,4-ethylene-dioxythiophene) (PEDOT) electropolymerization and formation of MnO_2_ NPs by redox exchange. This hybrid nanostructure can increase the transmittance of light. It has a high aspect ratio and high surface area, which makes the super capacitor superior transparency and excellent electrochemical performance and solves a big problem of transparent super capacitors [49]. Although the researchers involve a lot of effort on the transparent super capacitor, due to the limitations of current science and technology, the application of high performance transparent super capacitor has been hindered, which inspires us to put more enthusiasm on research of transparent super capacitor in the future.

### 3.3. Flexible and Transparent Super Capacitor

**Figure 5:**
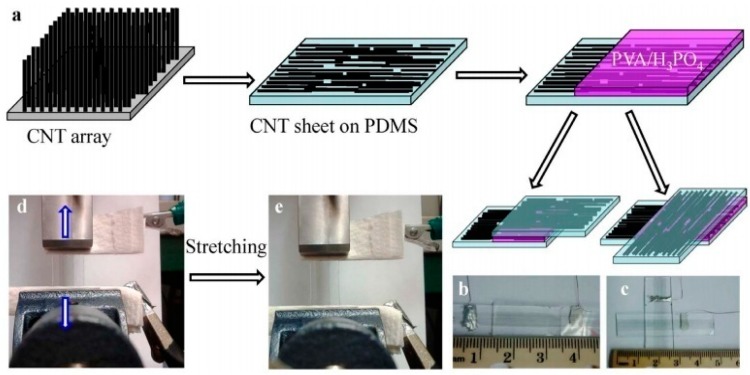
Diagram of the fabrication process for the transparent super capacitors and their optical images. (**a**) Schematic illustration of the process for fabricating the transparent and stretchable super capacitor. (**b**,**c**) Photographs of super capacitors assembled in the parallel and cross configurations. (**d,e**) Photographs of a super capacitor before and after stretching [66]. Copyright 2014, Nature Publishing Group.

As well known, the integrated advantages of high performance, flexibility and transparency super capacitor is widely studied [52,53,54,55,56,57]. Nevertheless, most existing super capacitors cannot be transparent and scalable at the same time, or it has these two characteristics but the electrochemical performance is not so good. Carbon nanomaterials are well suitable for flexible and transparent electronic devices. It is benefiting from their unique advantages, such as electronic and optical properties, structures, processability and compatibility [58,59,60,61]. One of application is the carbon nanotube based flexible and transparent layer [62,63]. Due to the high conductivity and mechanical property, carbon nanotube electrode has been reported frequently in the literature [64,65]. An important way to obtain flexible and transparent super capacitor with high performance is by aligning carbon nanotubes [64,66,67]. The aligned transparent and stretchable CNT film and their fabrication process are shown in Figure 5a. The assembled super capacitor in the parallel and cross configurations is in Figure 5b–e are photographs of a super capacitor before and after stretching [66]. The sample aligned with carbon nanotubes shows superior electrochemical performances than that of randomly dispersed carbon nanotube composite films, which makes its own great potential in application of novel flexible and transparent super capacitor. It can be seen that the material with higher alignment will have superior performance and also has higher transparency, high specific capacitance and longer life. It provides a new horizon for future transparent and flexible super capacitor. That is supplying new methods to improve material’s performance by precisely adjusting the structure.

**Figure 6:**
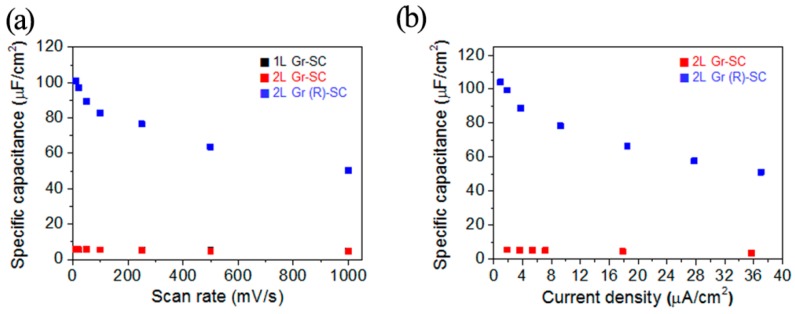
(**a**) The area-specific capacitances of 1L Gr-SC, 2L Gr-SC and 2L Gr (R)-SC calculated from the CV curves at the indicated scan rates. (**b**) The area-specific capacitances of 2L Gr-SC and 2L Gr (R)-SC calculated from the CD curves at the indicated current densities [71]. Copyright 2015, American Chemical Society.

Meanwhile, graphene also shows great potential in flexible and transparent super capacitor [68,69,70,71,72]. Graphene network is transferred onto a flexible and transparent polymer (e.g., PDMS) substrate. It shows both good optical transparency of 86% and mechanical flexibility [73]. In order to improve electrochemical properties and transparency of graphene electrode, a new strategy of stacked bilayer graphene and an ultrathin redox-active interlayer is invested. It can be clearly seen in Figure 6 that the electrochemical performance has been absolutely improved by this new strategy. Compare with normal graphene, this kind of super capacitor has almost 20 times at lower scan rates or 10 times at higher scan rates improvement in area-specific capacitance (in Figure 6a). Simultaneously, corresponding to the result in Figure 6a, the area-specific capacitance of the CD curve in Figure 6b is also increased. In addition, the super capacitor exhibits superior transparency of 75% and excellent flexibility [71]. This indicates that this redox-active interlayer is very stable between stacked bilayer graphene. It is of great significance to the generation of novel flexible transparent super capacitor.

Keunsik et al. fabricate the micro-super capacitor by chelating graphene quantum dots with graphene together. They attach graphene quantum dots to finger graphene by simple chemical deposition, and the graphene and graphene quantum dots form chelates through metal ions. The cross patterns of graphene form porous ipG-GQDs films. This kind of film exhibits high transparency of 92.97% at 550 nm, high energy storage of 9.09 μF cm^2^, and high stability even at severe bending angles of 45 degrees and 10,000 cycles. It indicates that the transparent and flexible interdigitated pattern of graphene-graphene quantum dot micro-super capacitor (ipG-GQDs-MSC) has strong potential for industrial integrated power supply [72]. Besides, faraday transition-metal-oxide/hydroxide (TMH) materials are often used to fabricate super capacitor. Na Li et al. realize the transparent micro-structured TMH electrodes formed by graphene enwrapped transition-metal-oxide/hydroxide (TMH) material, thus overcome the difficulties of preparation with the transparent micro-structured TMH electrodes. This microstructure can increase the speed of 3D electron/ion transport pathways, with a high specific capacity of 17.42 mF cm^−2^ at 0.2 mA cm^−2^ as well as a high capacity retention (85.1%) after 20,000 cycles [74]. Based on the above illustration, it can be seen that carbon nanomaterial based electrodes have great potential in achieving transparent and flexible super capacitors.

**Figure 7:**
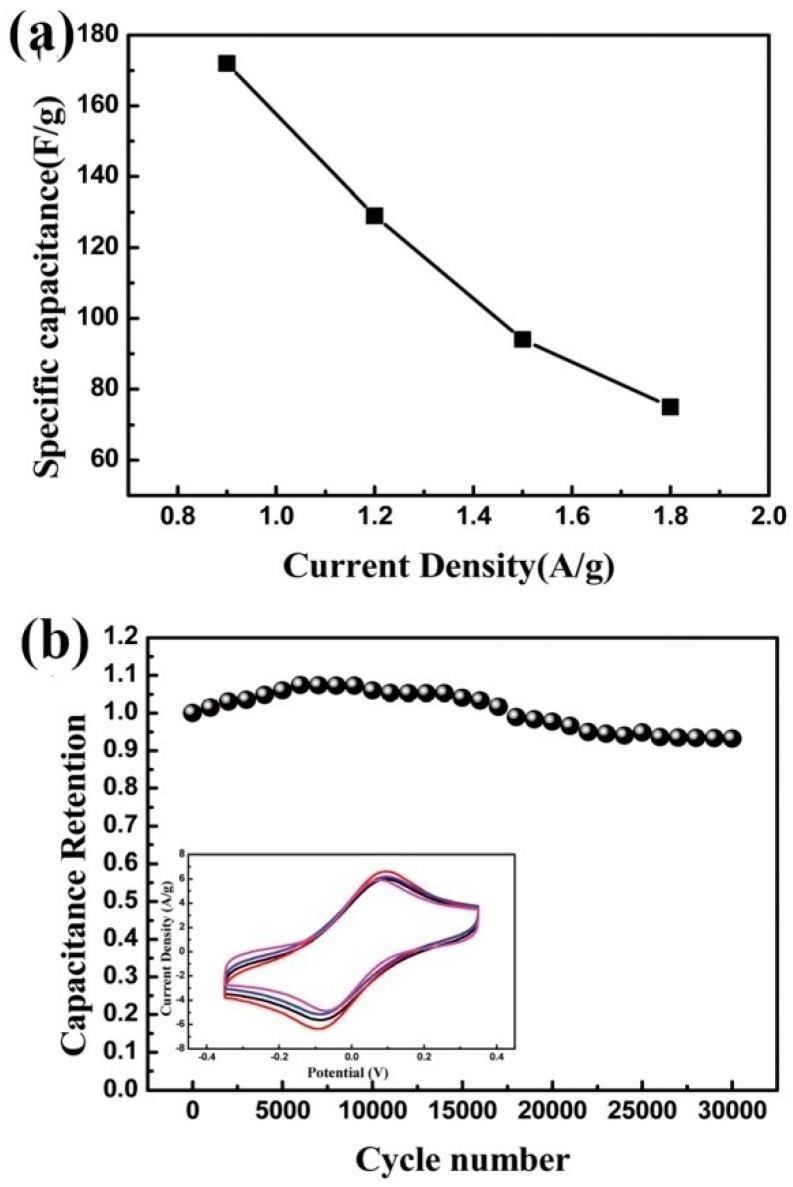
(**a**) Specific capacitance of the super capacitor at a series of current densities. (**b**) CV curves of the super capacitor at 10,000, 20,000 and 30,000 cycles, respectively [75]. Copyright 2016, the Royal Society of Chemistry.

Although carbon nanomaterials and graphene have broad applications in flexible and transparent super capacitor, they have many assignable drawbacks such as the poor or short cycle life, which makes them less mature in industry. Therefore, many other conductive materials are researched extensively to find out more suitable candidate for flexible and transparent super capacitor. Transition metal oxides have been widely used in the manufacture of super capacitor, with high electrical conductivity and excellent mechanical properties. The flexible and transparent Co_3_O_4_-based super capacitor exhibits superior property than that of carbon-based super capacitor. As shown in Figure 7a, the super capacitor gets a high capacitance of 177 F g^−1^ at a scan rate of 1 mV s^−1^. Especially, the capacitance retention achieves 100% after 20,000 cycles and 93% after 30,000 cycles (as shown in Figure 7b) [75]. Compared with the previously reported carbon-based pseudo capacitor, it has superior performance. It can largely solve the defects of super capacitors with short life. Moreover, an innovative method of laser ablation can synthesize ultrafine NiCo_2_O_4_ nanospheres. The transparent and flexible NiCo_2_O_4_-based device shows the cycling retention of 90.4% after 10,000 cycles. More importantly, this device gets a typical transmittance of 55% and shows an excellent mechanical flexibility, which means that transition metal oxides is an ideal active materials for transparent and flexible energy storage devices [76]. In addition, Ag nanowire is belonging to one of the most promising candidates for transparent and stretchable electronics. Compared with typical Ag nanowire device, a modified Ag-Au core-shell nanowire percolation network electrode can greatly improve the electrochemical instability with the stable performance up to 60% [77].

**Figure 8:**
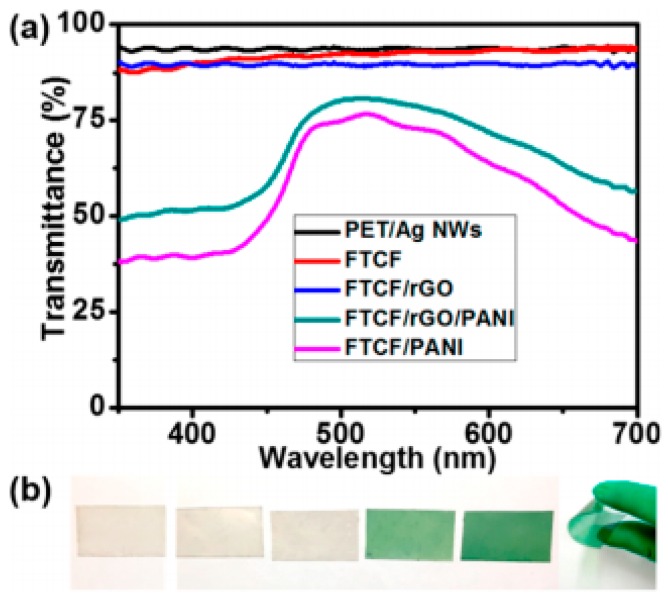
(**a**) Transparency spectra of PET/Ag NWs film, FTCF substrate, FTCF/rGO, FTCF/rGO/PANI and FTCF/PANI, respectively. (**b**) The photographs of corresponding film from (left to right) for PET/Ag NWs film, FTCF substrate, FTCF/rGO, FTCF/rGO/PANI, FTCF/PANI and the flexible FTCF/rGO/PANI [79]. Copyright 2017, American Chemical Society.

PANi nanowires-based electrode attracts much attention of researchers due to the high specific capacitance, low monomer cost and relative flexibility [78]. According to the above, graphene is the competitive material candidates of super-capacitor electrodes. Fanhong Chen and co-workers fabricate nanocomposite films of reduced graphene oxide (rGO) and aligned polyaniline (PANI) to integrate the advantages of these two materials. The device shows excellent transparency in Figure 8. The transmittance of film is gradually decrease from top to bottom that 93.67% for PET/Ag NWs film, 92.96% for FTCF substrate, 89.67% for rGO-coated FTCF film (FTCF/rGO), 78.76% for FTCF/rGO/PANI and 72.92% for PANI-coated FTCF film (FTCF/PANI) at 550 nm, respectively, as shown in Figure 8a. And the corresponding film form from left to right is shown in Figure 8b. It can be also known that the FTCF/rGO/PANI nanocomposite film has excellent bendability [79]. The assembled super capacitor exhibits excellent capacitance performance, enhanced transparency and superior flexibility because of the synergistic effects of PANI and graphene, the growing electron transport from graphene and the large surface area from aligned PANI film promoting ion diffusion. This method attempts a wonderful strategy of synergistic effect to promote the development and application of flexible and transparent super capacitor.

## 4. Conclusions

With the rapid progress of electronic technology, it seems that many researchers are full of beautiful longing for comfortable and multi-function wearable devices. The energy supply equipment is one of the most important problems to be solved. Among the energy storage systems, lithium-ion batteries and super capacitors are important components of the device, integrating transparent and stretchable properties into an energy storage device which restricts the development of these electronic devices is a great challenge. In recent years, great progress has been made in materials exploration, structural design, manufacturing methods and integrated assembly of flexible and transparent energy storage devices. Flexible and transparent energy storage devices can be implemented in two directions: New structure design and new material exploration. Herein, the development of flexible and transparent lithium-ion battery and super capacitors has been introduced in the paper. Under persistent efforts, many new materials and new strategies are applied in lithium-ion battery or super capacitor. Carbon nanomaterial like carbon nanotube, carbon nanofiber and graphene and the new design of these materials have been adopted to develop energy storage systems. Carbon nanotube is usually used as supporting part, which can improve the electronic conductivity. But, the electronic property of carbon nanotube is comparatively lower than that of graphene. Moreover, graphene with large specific surface area can effectively alleviate the volume expansion in the process of lithiation, which makes it a promising candidate for future flexible and transparent storage devices. The application of other conductive materials in energy storage devices, such as transition metals and transition-metal-oxide/hydroxide (TMH) material, is also introduced. However, due to various factors, the development of these materials in flexible transparent lithium-ion battery and super capacitor cannot meet the needs of practical applications. Thus, in the future, it is very important and desirable to enhance property of flexibility, transparency and electrochemical of energy storage device. Of course, there are many problems and challenges to realize the practical application of flexible and transparent energy storage devices, such as to explore low-cost material, to improve the cycle stability of flexible devices, to improve the large-scale production technology and so on. That requires researchers to devote more energy to electrode material research, electrode structure design and production process development. On the other hand, we believe that, with the rapid development of science and the continuous efforts of scientific researchers, this kind of energy storage device with high performance and special functions will be realized in the near future, which will greatly improve the quality of our life.
